# Striving for Racial Equity in Oral Cancer Research: A Case Study

**DOI:** 10.5694/mja2.70238

**Published:** 2026-07-01

**Authors:** Sneha Sethi, Simon Naylor, Catherine Leane (Dharug/Gabrigal), Gail Garvey (Kamilaroi), Joanne Hedges (Yamatji), Lisa M. Jamieson, Nicolas Reid (Dharug/Gabrigal)

**Affiliations:** ^1^ Adelaide Dental School Adelaide University Adelaide South Australia Australia; ^2^ Aboriginal Health Division Women’s and Children’s Health Network North Adelaide South Australia Australia; ^3^ School of Public Health The University of Queensland Herston Queensland Australia

**Keywords:** cancer, clinical competence, indigenous health, racism

## Abstract

Racism impedes the achievement of equity in healthcare by permeating individual, community, societal and institutional levels. Cancer is the leading contributor to global mortality and continues to have a disproportionately higher impact on First Nations Peoples. Research specific to First Nations Peoples, conducted in accordance with the principles of Indigenous research, is critical to justify the advocacy and delivery of measures that yield relevant, translatable outcomes and benefits. The following article presents a case study of a longitudinal cohort project assessing human papillomavirus‐associated oral cancer in First Nations Peoples of South Australia. The article discusses decolonising methodologies, their application and relevance.

## 1 Background

Racism is identified by biased intentions, judgements and actions at the individual, institutional and systemic levels, which are supplemented by the urge to oppress, stemming from feelings of superiority, accentuating power differentials [1]. A racist landscape favours prejudiced social outcomes and health inequities [2, 3]. A distinct appreciation of race as contributing to health inequalities has facilitated meditative, racially influenced, equitable health research [2, 4, 5], causal and intersectional analyses [6, 7] and the encouragement of culturally safe, unbiased care within the medical community. Health systems can create pathways to alleviate discriminatory health outcomes by exploring the convoluted relationships between race, social drivers of health and prejudiced health inequities [4]. Intentional measures to promote an anti‐racist culture after acknowledging racist behaviours are essential to accomplish an open‐minded, impartial and competent public health system. Clinical research (observational, cross‐sectional, longitudinal), trials and other forms of academic research are critical in providing an evidence base to inform public health initiatives and treatment guidelines.

Clinical research is conducted with the primary aim of acquiring deeper knowledge of a disease, its biology and the efficacy and toxicity of treatment strategies, thereby informing management decisions. However, achieving diversity in clinical research is challenging. Certain factors with established effects on disease impact, such as age, sex and ethnicity, are insufficiently understood and underrepresented in clinical research. This leads to the development of strategies that are incapable of addressing the needs and specifications of vulnerable (minoritised and racialised) populations, depriving them of equitable quality of care. Thus, reciprocal, collaborative, unbiased, culturally safe and inclusive research practices are non‐negotiable for achieving equitable, translational outcomes in clinical research.

This article highlights insights and knowledge gained over the course of a longitudinal cohort clinical research study. Grounded in principles of decolonisation, this case study employs a racial equity lens to develop culturally safe, unbiased clinical research practices that aim to eliminate racial disparities and improve outcomes.

## 2 Our Positionality

As a team of First Nations and non‐Indigenous individuals, we acknowledge and respect the honour of working for and alongside First Nations Communities across Australia. Under First Nations leadership (JH, NR) and governance (CL, GG), this team has worked over the past decade to achieve racial equity in oral health and oral cancer research. Our research is guided by Indigenist methodologies grounded in our commitment to equity and impartiality. We recognise our personal biases and prioritise self‐reflexive sharing of emotions and stories, which helps overcome these opinions and judgements. This team comprises clinical experts (NR, SN, SS, LJ) who, through ongoing processes of reflexivity, continually remind one another of the positions of power and privilege they may project.

## 3 The Cohort

First Nations People are the *First* peoples of Australia; they are among the longest‐surviving and most resilient populations in the world [5]. First Nations Peoples have survived decades of sustained intergenerational trauma, persistent racialisation through cultural assimilation and personal annihilation policies [6, 7]. But the consequence of these stringent policies and continual discrimination has had distressing impacts on the social, emotional wellbeing and health of First Nations Peoples in Australia [8]. First Nations Peoples in Australia have a higher incidence of, and lower 5‐year survival rates from, cancer compared with the non‐Indigenous population [9]. Specific data pertaining to human papillomavirus (HPV) infection‐associated oropharyngeal cancers is sparse, although a substantial increase in the incidence rate of head and neck cancers in First Nations People has been reported [10].

The Indigenous Australian Human Papillomavirus Cohort Study (HPV‐OPC project) is a prospective longitudinal cohort study developed in partnership with Aboriginal Community‐Controlled Health Organisations (ACCHOs), local First Nations communities in South Australia and First Nations health researchers; funded by Australia's National Health and Medical Research Council [11, 12]. The cohort was established in 2018 and has been followed up annually since recruitment. The study is governed by an Aboriginal Reference Group, with data collected by trained First Nations research officers. The Aboriginal reference group comprises all Aboriginal experts in health, research, dentistry, medicine, public policy, ethics and cultural safety. It is chaired by a senior Aboriginal health researcher with rich cultural and health research experience. The primary aim was to evaluate population estimates of HPV in the mouth and oropharynx among First Nations People of South Australia. The 1011 participants recruited at baseline represented 5% of Aboriginal and/or Torres Strait Islander South Australian adults eligible during the recruitment period.

## 4 Lessons Learnt

### 4.1 Robust Community Engagement

Building a strong foundation with the community before commencing any research project is essential for establishing shared power and decision‐making grounded in reciprocity and mutual benefit. In the HPV‐OPC project, a strong First Nations partnership and engagement have been described against the Consolidated Criteria for Strengthening the Reporting of Health Research involving Indigenous Peoples (CONSIDER) statement [13] (Supporting Information S1) [14].

Specific steps undertaken to ensure robust community relationships included First Nations‐led comprehensive community consultations conducted before project initiation to ascertain community‐identified priorities; a consistent emphasis on First Nations capacity building within the team; and oversight by an established reference group at all times. The development of oral cancer prevention resources warranted co‐design and partnership with First Nations stakeholders, patients, doctors and community members to embed First Nations voices and knowledge. Strong relationships with the community are also paramount for retention in a longitudinal cohort study, as completing follow‐ups ensures timely oral cancer screening, education and prevention. First Nations leadership and data sovereignty practices establish trust in the research team, which is key to achieving strong relationships and translating benefits to a broader community. For future clinical studies involving First Nations Peoples and communities, it is highly recommended to establish robust community engagement before, during and after the study. This approach ensures the prioritisation of community‐identified needs and the realisation of tangible benefits for the communities involved.

### 4.2 Not Everything Will Go as Planned; Sometimes Nothing Will

Western research methodology entails rigid linear frameworks and strict adherence to timelines and protocols, which create an intimidating power differential between the researcher and the participant. Similarly, clinical research is precise and requires consistency in recording and reporting to avoid discrepancies in results that inform treatment guidelines, public policy reports or recommendations for screening and disease prevention. To create a culturally responsive research environment, it is crucial to balance rigid clinical research directives with an adaptive methodological approach while diminishing power imbalances. Oral cancer screenings and clinical examinations with the cohort allowed the team to realise this flexibility while maintaining scientific rigour. The process was visualised as cyclical and iterative, encompassing discussion, refinement and adaptation. These adaptations and discussions were often led and inspired by engagements with participants, First Nations researchers, ACCHO staff, Aboriginal health workers and stakeholders. For example, the oral examination component of the study was exhaustive, tedious and tiresome for participants and laborious for the clinician. This module was subsequently simplified into a faster, more concise version after discussions with participants and senior researchers. Participants described the process of oral examinations as intimidating, reporting feelings of vulnerability while positioned in the dental chair with their mouths open for extended periods. This feedback was incorporated into the study design, with participants given the option to undergo dental examinations from the comfort of their homes, on their couches surrounded by family, on verandahs or under the shade of a tree, often with curious children and pets present. This flexibility ensured a holistic research framework with a methodology grounded in decolonial principles, aligned with First Nations values and knowledge systems and respectful of Indigenous space and time. Collaborative yarning was one of the Indigenist research practices [15] utilised to revisit and adapt our clinical research approach, thereby fostering a respectful, empathetic and balanced relationship with the cohort and privileging the knowledge and voices of First Nations People. For future clinical studies, it is imperative to allow flexibility in methodologies and frameworks, thereby fostering a culturally responsive and inclusive environment. This approach will be crucial for sustaining enduring relationships with participants and their families, as well as enabling unencumbered data collection.

### 4.3 Take a Moment to Listen

Researchers and clinicians operate from learned behaviours/assumptions of greater knowledge and strength, often overlooking the participant's perspective. Deep listening or ‘Dadirri’ is an Indigenist methodology centring on First Nations voices involving conscientious, vigilant, reciprocal sharing of knowledge, which helps build confidence and trust in the researcher‐participant relationship. This methodology ensures that the researcher is better informed about the participant's situation and narrative styles, is cognizant of personal circumstances, avoids interrupting and is respectful of the line of questioning. This is yet another approach to reducing power imbalances between the researcher and the participant and to fostering an appreciative, humble, two‐way knowledge exchange in a culturally appropriate manner. For example, many times researchers were informed how the participants felt conscious of their teeth, shared a personal story related to a previous traumatic dental visit or referred to the shame they felt with their teeth. These sharings helped the researcher utilise an indirect line of questioning or motivate the participant about their oral health and facilitate a healthy discussion, devising tangible outcomes and strategies that could be beneficial to participants, their families and communities. Avoiding interruptions and encouraging active, deep, attentive listening fostered a positive relationship between the researchers and participants, in which participants felt comfortable undergoing a clinical examination and sharing their fears about oral cancer. The participants felt heard and respected and were receptive to the oral cancer prevention and screening information provided by the researchers. A level of trust is essential for achieving favourable outcomes in a culturally safe and appropriate manner, but it requires substantial time and commitment from the researcher. In future clinical studies, integrating this lesson into trials will yield essential insights concerning patient behaviour and response, thereby fostering trust.

### 4.4 Leave Your Assumptions and Clinical Jargon at Home

As researchers and clinicians, we often conduct our research from personal standpoints and worldviews. It is therefore critically important to situate oneself within the research from the participants' perspective. The researcher operates and assumes from the position of privilege, power and rigid Western knowledge‐based systems, whereas the participant has often faced a lifetime of judgement, discrimination and disadvantage based on race. It is important to identify personal biases and to repeatedly acknowledge the power differential between researchers and First Nations participants. This involves continuous reflexivity activities with Indigenous researchers and other team members; to reflect on one's individual positionality and the impacts this might have on the research process, relationships and outcomes. The use of clinical jargon reinforces a power differential, which is likely to threaten engagement and reduce participants' trust in the researcher, potentially impeding the extent of information that participants retain. Clinical yarning [16] has been identified as a culturally safe method for conducting clinical research with First Nations communities, involving a storytelling process [17] in which participants are encouraged to share personal stories and reflections, allowing sensitive issues to emerge at their own pace. These steps, away from a fixed, rigid questionnaire, guide the researcher to attend to cues that inform the clinical examination or research questions. Encouraging the incorporation of clinical yarning techniques supports the avoidance of clinical terminology and makes the researcher more aware of their language and approach with the participant. This entire process involves rigorous training for the research team and multiple iterations, with the aim of reintroducing new lines of questioning and greater flexibility within clinical research. For example, when researchers learned about the fear and stigma associated with the word ‘cancer’ in communities [18], this led to the project subsequently being referred to as the ‘throat sickness project’ to alleviate fear and avoid unnecessary panic. Another example of clear, healthy and transparent communication with participants was practiced while performing clinical examinations; the researcher called out numbers such as 0, 1 or 2 to code for specific findings in the mouth and throat, which was confusing and scary for the participant, so the researchers/clinicians mandated a conversation before and after examination discussing all the findings and what each code meant in order to foster a transparent research practice, align with Indigenous data sovereignty principles [19], as well as strengthen relationships and trust with the participants and their families. Discussing these codes and the relevance of the numbers beforehand encouraged a more relaxed examination atmosphere, and a discussion after the examination also created the opportunity for follow‐up questions from the participants. All efforts were made to alleviate participants' apprehensions and to translate the individual oral findings for each participant, thereby reinforcing self‐determination. Future clinical studies must regard this lesson as an indispensable principle during the execution of trials; power imbalances should be eradicated, and research or clinical teams are expected to strive towards ensuring the highest standards of cultural safety.

### 4.5 We Are the Ones Learning, Not the Other Way Around

Conducting unbiased, respectful and beneficial research entails ‘un‐learning’ [17]. What has been learnt? This does not mean that prior findings are wrong; although clinically coherent and correct, they are of little benefit in Indigenous health research. The knowledge base systems regarding health, healing and wellbeing are far superior and different; the health support frameworks are holistic and address all aspects of a person's wellbeing (physical, social, emotional, mental and spiritual). Data cannot be collected on paper under strict headings; it is a more time‐consuming, collective, iterative process. The process of clinical research involved dismantling colonial ideologies, stepping away from Westernised perceptions, removing whiteness supremacy dialogue and highlighting Indigenous ways of knowing, being and doing. A clinician conducting research often must resist the natural urge to provide advice or treatment, as the research process is limited to observation and analysis. Learning from integrated, culturally rich communities and participants, the research team went beyond standard protocols and provided support through affirmation, oral hygiene instructions, assistance with scheduling dental appointments, and, on participants' behalf, reporting instances of racial misconduct in dental clinics. The researchers took time to understand Indigenous ways and to respect each distinct community and its traditions. All clinical studies must allow room for this un‐learning process, which involves critical, reflexive activities with the team moving away from dominant white perspectives and methodologies.

### 4.6 Acknowledge the Privilege

The destructive impacts of colonisation have been indisputable over the past two centuries, resulting in socioeconomic deprivation and health inequities. In the face of these impacts, a pervasive attempt to recognise and endorse Indigeneity demonstrates respect for the traditional custodians of the land [20]. Although pivotal to First Nations Peoples and Communities, this practice is also political, which ‘invokes pride that Australians are learning the name of the First Nation Lands upon which they were born or arrived, or live and work’ [18]. Conducting research on stolen lands with and for Indigenous People should be rooted in gratitude, respect, reciprocity, reflexivity, honour and sincerity. Thus, highlighting the importance of conducting research with Communities and incorporating co‐design principles from the very beginning. Including mandatory First Nations researchers and voices in the research ensures respectful and culturally appropriate outcomes. Knowledge of the Traditional Owners and true custodians of the country should be taught to team members, with a mandate to acknowledge Country and its People before initiating any research. This fosters a sense of humility among team members and ensures that the research is guided by Indigenous values rather than by pockets of privilege. This practice is usually delivered before meetings or events, or in communications, but incorporating it before starting any research project provides an opportunity to show respect for the Traditional Owners, recognise the continuing cultural and spiritual connection, and wear Aboriginal‐designed uniforms and have Indigenous signage on vehicles. Maximising the delivery of this acknowledgement prevents tokenisation of the gesture and enriches the delivery of research outcomes. While working on the HPV‐OPC project, all team members were guided by Aboriginal leaders and Aboriginal research officers to deliver an Acknowledgement of Country at each site upon arrival. This facilitated the grounding of the team members' energies and intentions in Indigenous values and principles and instilled a sense of obligation to conduct the research from an unbiased standpoint. This lesson may prove especially beneficial for future clinical studies, as it helps anchor resources and purposes within Indigenous beliefs, fostering a sense of duty to conduct the research impartially.

### 4.7 Changing the Narrative

Dominant white colonial structures and power differentials have dictated a deficit‐focused narrative for discussing, reporting, exploring and analysing outcomes of research on First Nations Peoples [21]. A decolonised approach to this practice endorses shifting the narrative to a strengths‐based approach [22], embedding Indigenous knowledges, ingraining Indigenist methodologies such as yarning, deep listening and storytelling and reinforcing self‐determination [23]. This shift can be fostered by refuting negative stereotypes, supporting data sovereignty and highlighting strengths, resilience, rich culture and robust knowledge‐based systems. This adaptation is imperative in research to abate the impacts of harmful acts, policies and behaviours, amplify Indigenous voices and warrant research that supports community‐identified priorities. The HPV‐OPC project is delivered by a mix of Indigenous and non‐Indigenous students, staff and research officers involved in data collection, analysis and reporting. Indigenous leadership in the project ensures ongoing capacity building for other Aboriginal staff and students and affirms self‐determination. Contrary to historical practices, the team now ensures the delivery of research outcomes (publications, reports, presentations) through a strengths‐based narrative, shifting the focus from the burden of disease to the cohort's resilience. This step represents a crucial phase in the continuous learning process for all existing and future teams; it will require a significant, intent‐driven effort but will ultimately help mitigate the impacts of harmful acts, policies and behaviours.

### 4.8 Data Sovereignty

First Nations data sovereignty asserts the fundamental right of First Nations Peoples to have complete control over their data, including data collection instruments, priorities, collection, analysis, reporting and management [24]. The HPV‐OPC project has been governed by the Indigenous Oral Health Reference Group, a working group of First Nations researchers, leaders and community members established in 2011. This encourages input from First Nations researchers on quality control, data collection methods and instruments and helps ensure that the research continues to support the priorities of First Nations Peoples, communities and organisations. Data collected in this project have been based on community consultations and on needs identified by participants [22, 25]. While performing oral cancer scans, many questions related to prevention and early signs were asked by the participants, following which culturally appropriate resources for the Community related to oral HPV infections and oral cancers were co‐designed and disseminated. Data sovereignty principles mandate free and prior informed consent from participants. The HPV‐OPC project obtains written consent from participants before each follow‐up, and a separate consent form is obtained for the oral cancer examination. This ensured that participants understood the study's components and retained control over their participation. After completion of each follow‐up, newsletters summarising the collected data and reports were sent to the local ACCHOs and participants. Data collected after oral cancer screens and oral HPV infection status have been provided to all the participants, and any high‐risk participants with persistent infection with a high‐risk HPV strain have been made aware of the risks and potential of developing a malignant lesion. Subsequently, the team facilitated appointments with doctors, dentists and oral cancer specialists to ensure a culturally safe experience for the participants. Future clinical studies must incorporate this lesson into practice, encompassing informed consent, a clear, coherent and concise understanding of the study and transparent data findings.

## 5 Conclusion

Health equity mandates the establishment of guidelines that address systemic barriers, rigid structures and the increased burden of disease to create opportunities for each participant to be as healthy as possible. To the best of our knowledge, the HPV‐OPC project is one of the most successful longitudinal cohort studies in the world, with a focus on oral HPV infections and oral cancers among a First Nations community. Steps have been taken from the early planning stages through to the final delivery of outcomes, ensuring benefits for the community, sustainable approaches, cultural safety, community leadership and effective screening for oral cancers. These learnings can be adapted for use in clinical research projects involving First Nations Peoples to achieve equitable outcomes.

## Author Contributions

**Sneha Sethi:** conceptualisation, data curation, formal analysis, investigation, methodology, project administration, validation, visualisation, writing – original draft, writing – review and editing. **Simon Naylor:** conceptualisation, investigation, validation, visualisation, writing – review and editing. **Catherine Leane (Dharug/Gabrigal):** investigation, methodology, project administration, supervision, validation, visualisation, writing – original draft, writing – review and editing. **Gail Garvey (Kamilaroi):** conceptualisation, funding acquisition, investigation, supervision, validation, visualisation, writing – review and editing. **Joanne Hedges (Yamatji):** conceptualisation, funding acquisition, investigation, supervision, validation, visualisation, writing – review and editing. **Lisa M. Jamieson:** conceptualisation, funding acquisition, investigation, supervision, validation, visualisation, writing – review and editing. **Nicolas Reid (Dharug/Gabrigal):** conceptualisation, funding acquisition, investigation, supervision, validation, visualisation, writing – review and editing.

## Funding

This work was supported by the National Health and Medical Research Council (NHMRC) project grant (APP1120215). Gail Garvey (Kamilaroi) is supported by an NHMRC investigator grant (APP1176651). Lisa M. Jamieson is supported by an NHMRC research fellowship (APP1102587).

## Disclosure

Not commissioned; externally peer reviewed.

## Conflicts of Interest

The authors declare no conflicts of interest.

## Supporting information


**Data S1:** CONSIDER statement.

## Data Availability

No data was generated for this paper, given the narrative nature of the script. Any findings discussed have been appropriately referenced in the manuscript.
