# Supplementary material for: Striving for Racial Equity in Oral Cancer Research: A Case Study
Source: Med J Aust. 2026 Jul 1;224(7):e70238. doi: 10.5694/mja2.70238 (PMC13323945; doi:10.5694/mja2.70238)
Supplement: Supplementary file 1 — Data S1: CONSIDER statement. [file MJA2-224-0-s001.pdf]

The Medical Journal of Australia

Supporting Information

Supplementary material

This appendix was part of the submitted manuscript and has been peer reviewed.
It is posted as supplied by the authors.

Appendix to: Sethi S, Naylor S, Leane C, et al. Striving for racial equity in oral cancer research: A Case Study. *Med J Aust* 2026; doi: 10.5694/mja2.70238.

CONSIDER Statement Template

Guest Editors of the 2026 *Indigenous Health Special Issue* acknowledge the Indigenous expertise that informed the establishment of the CONSolIDated critERtia for strengthening the reporting of research involving Indigenous Peoples (CONSIDER) statement.

Authors should indicate how they have supported ethical publishing and reporting practices by providing the details of the research practices aligned with this publication in accordance with the CONSIDER statement. The reporting should not exceed two pages. This reporting will be published as online supplementary information. Detailed items can be accessed in the publication:

https://bmcmedresmethodol.biomedcentral.com/articles/10.1186/s12874-019-0815-8

**Governance**
Formal letters of support from participating Aboriginal Community Controlled Health Organisations (ACCHOs), Harm minimisation included as part of informed consent processes for all phases of the study, the Aboriginal human research ethics requirements, Protection of Indigenous intellectual property and knowledge emphasised in each of the community consultation and engagement sessions, and individually with participants through the informed consent process.
**Prioritization**
The idea for the study was first articulated by Indigenous community members following a feedback session on another health research project conducted by the study investigators. Extensive community engagement and consultation to refine the study aims.
**Relationships (Indigenous stakeholders/participants and Research Team**
Ethics approval was sought and obtained from two separate human research ethics committees: the University of Adelaide and the Aboriginal Health Council of South Australia.<br>Initial consultations with all interested ACCHOs, Funding application submitted that reflected Indigenous community views and inputs in study design and methodology, 12 Indigenous investigators included, community consultation repeated once funding was received, with ACCHOs providing structures, strategies and recommendations for recruitment and data collection., Staff hired (5 Indigenous, 2 non-Indigenous), with non-Indigenous staff undertaking cultural competency training, An Indigenous Reference Group established to provide oversight and cultural guidance. 12 Indigenous investigators, all recognised leaders in their respective Indigenous health research fields, 7 of the 12 non-Indigenous research team extensive experience working with Indigenous communities
**Methodologies**
Large scale observational study with follow-up after 12, 24, 48 and 60 months, All data de-identified, with field staff not analysing data once entered in database, Baseline questionnaire (as consequence of community consultation) included items pertaining to experiences of racism, major life events (incarceration, death, child removal), social disadvantage and access to health services, sleep, dental service utilisation, resilience and food security.
**Participation**
Consent forms explicitly stating that no third parties will have access to samples or data, Any secondary analyses/long-term follow-up of study participants will only be conducted by research team, Participating ACCHOs provided support only when their resources allowed, Participants were made aware, during the informed consent process, of the time commitments to being involved in the study, Saliva samples genotyped for HPV once and then destroyed, Data stored on password-protected computer software at the University of Adelaide for 15 years

**Capacity**
Study employed five Indigenous staff (one the project manager), Indigenous research assistants, including those volunteered by ACCHOs, trained in research skills, ethics principles, data collection, data checking, data filing and disseminating research findings back to community, ACCHO staff given opportunity to represent the study at national and international meetings, ACCHOs and study participants were able to bolster their knowledge of HPV infection and OPSCC, and the links between the two, through free and frank conversations with the research team, Knowledge-sharing was two-way, with substantial benefits for the non-Indigenous research staff in being included in Indigenous consultative processes and learning from ACCHOs and study participants**.**
**Analysis and interpretation**
Research analyses have included all key Indigenous stakeholders and Indigenous researchers as co-authors on publications. This has enabled Indigenous values and perspectives to inform the interpretation of the study's findings. All papers and presentations ensure a strengths-based narrative.
**Dissemination**
Presentations to key Indigenous stakeholders and other Indigenous community groups, presentations at international conferences (Indigenous project manager and two ACCHO staff), The findings will hopefully support increased resourcing for Aboriginal Health Workers to be specifically employed to facilitate increased understanding of the links between HPV and OPSCC This will, in turn, increase capacity in other areas of HPV research, including translating to policy for screening for HPV-related oral cancers. Dental findings were immediately relayed to participants with supportive resources provided. Knowledge and awareness-based resources have been co-designed with Indigenous team members and community members regarding HPV infections and oral cancer. All resources have been circulated to ACCHOs across South Australia and to other relevant health organisations, such as the Royal Flying Services.
